# Analyzing pathogenic variants in mismatch repair genes: personalized prevention strategies for lynch syndrome in Chinese families

**DOI:** 10.3389/fmed.2025.1527249

**Published:** 2025-03-14

**Authors:** Xiufang Wang, Haichun Ni, Lin Zhu, Hui Huang, Aiping Deng, Jifa Hu, Wei Cai, Juyi Li

**Affiliations:** ^1^Department of Pain, The Central Hospital of Wuhan, Tongji Medical College, Huazhong University of Science and Technology, Wuhan, China; ^2^Department of Pathology, The Central Hospital of Wuhan, Tongji Medical College, Huazhong University of Science and Technology, Wuhan, China; ^3^Department of Pediatrics, Tongji Hospital, Tongji Medical College, Huazhong University of Science and Technology, Wuhan, China; ^4^Department of Gastrointestinal Surgery, The Central Hospital of Wuhan, Tongji Medical College, Huazhong University of Science and Technology, Wuhan, China; ^5^Department of Pharmacy, The Central Hospital of Wuhan, Tongji Medical College, Huazhong University of Science and Technology, Wuhan, China; ^6^Department of Scientific Research, The Central Hospital of Wuhan, Tongji Medical College, Huazhong University of Science and Technology, Wuhan, China

**Keywords:** lynch syndrome, mismatch repair gene, whole-exome-sequencing, Sanger sequencing, genetic counseling

## Abstract

**Background:**

This study aimed to analyze the pathogenic variants in one family with colorectal cancer and another with endometrial cancer and provide appropriate personalized prevention strategies for carriers of these genetic mutations.

**Methods:**

One proband with colorectal cancer and another with endometrial cancer and their family members were enrolled in this study. Whole-exome sequencing was used to identify pathogenic gene mutations in both families. We compared the structural difference between the wild-type and mutant MSH2 proteins using SWISS-MODEL and PyMOL visualization software.

**Results:**

We identified one novel mutation (NM_000251.2:c.1486delT:p.L496*) in the *MSH2* gene in Family I and a known mutation (NM_001258271.1:c.884 + 4A > G) in the *MLH1* gene in Family II. The novel mutation (NM_000251.2:c.1486delT:p.L496*) caused a stop gain mutation, resulting in the absence of amino acids 496–934 in the mutant MSH2 protein. This led to the loss of Domain 5 and alterations in the sequences of Domain 3 and Domain 4 regions, resulting in premature termination of MSH2 protein coding. The known mutation (NM_001258271.1:c.884 + 4A > G) in *MLH1* causes the skipping of exon 10, producing a truncated protein and undergoing nonsense-mediated decay based on literature reports. Thus, 5-fluorouracil-based adjuvant chemotherapy is not recommended for patients with lynch syndrome

**Conclusion:**

The novel stop gain mutant (NM_000251.2:c.1486delT:p.L496*) in *MSH2* is deemed pathogenic for LS, and the mutant (NM_001258271.1:c.884 + 4A > G) in *MLH1* has been further confirmed to be pathogenic. These findings expand the spectrum of mismatch repair gene variations in the ethnic group Han of China and reaffirm the importance of genetic testing for LS.

## Introduction

Lynch syndrome (LS), also known as hereditary nonpolyposis colorectal cancer, may be the most common inherited cause of susceptibility to cancer, with an incidence rate ranging from 1 in 100 to 1 in 180 (1). Individuals with LS are prone to various cancers, the most common of which are colorectal and endometrial cancers. They are also at risk for cancers in many other organ sites, including the stomach, small intestine, ovaries, prostate, and skin (sebaceous glands) (2, 3).

LS is a hereditary, heterogeneously autosomal dominant disorder caused by pathogenic variants in mismatch repair (MMR) genes, including *MSH2*, *MSH6*, *MLH1*, and *PMS2* (4, 5). Variations in these genes disrupt the MMR process, leading to changes in the length of DNA microsatellite repeat sequences and the emergence of microsatellite instability (MSI), which accelerates the accumulation of somatic mutations and thus, accelerates tumor formation (6). Therefore, individuals with these genetic variations are more likely to develop cancer compared to the general population and often develop cancer earlier (7).

Therefore, it is crucial to identify the mutational profile associated with LS in the Chinese population to allow the implementation of oncogenetic counseling based on genetic tests specific to this population. In this study, we report a novel *MSH2* variant and a known *MLH1* variant in a Chinese family with colorectal cancer and another family with endometrial cancer, respectively. This study aimed to provide genetic counseling to the family members and to develop appropriate prevention strategies and precise treatment plans for individuals carrying these mutations.

## Methods and materials

### Participants

Two families (Han nationality), one affected by colorectal cancer and the other by endometrial cancer, were recruited from the Central Hospital of Wuhan. The diagnostic criteria for LS were based on a combination of the Amsterdam II criteria, clinical test reports, and detailed family pedigrees. This study was approved by the Ethics Committee of the Central Hospital of Wuhan. Informed consent was obtained from all participants involved in the study.

### Participants’ clinical characteristics

**Figure 1:**
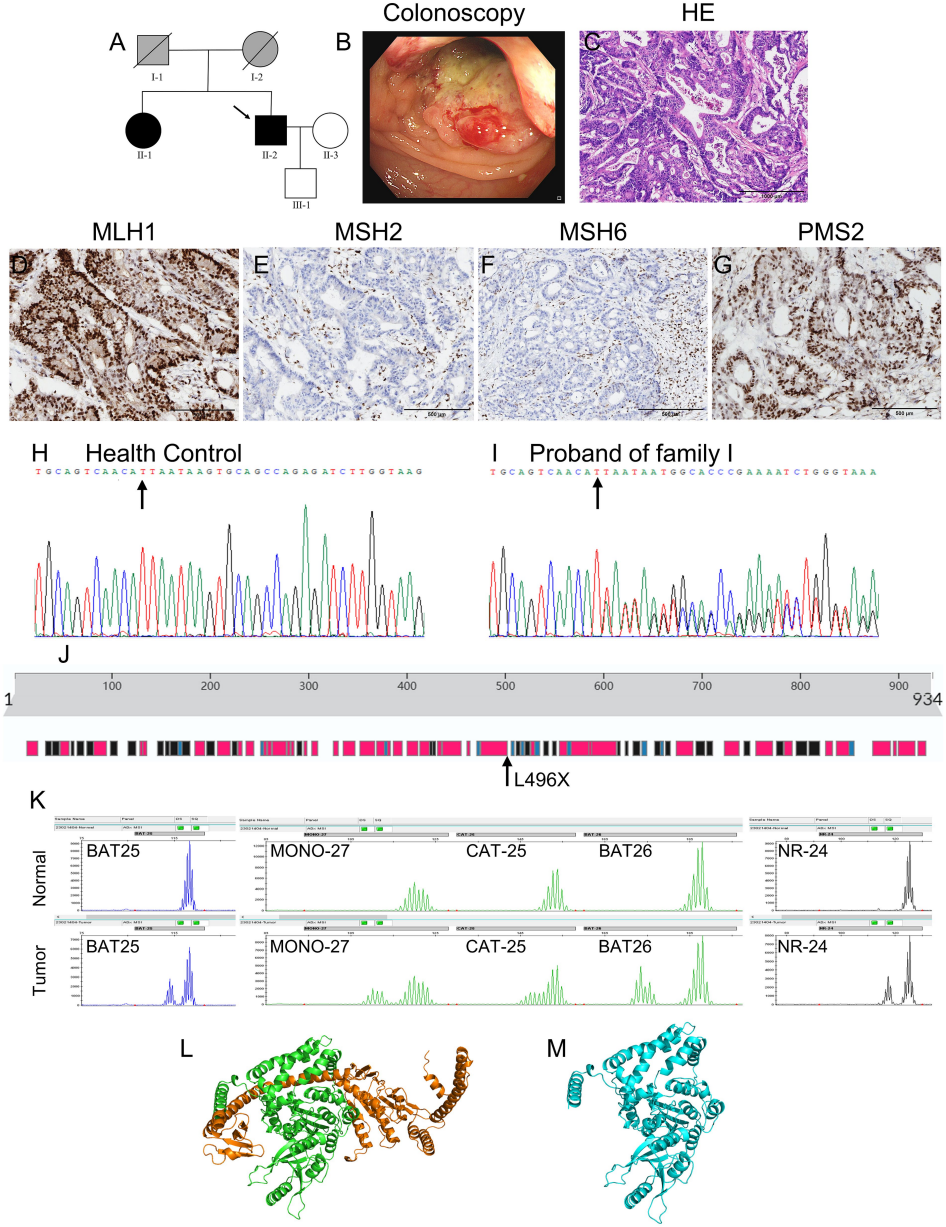
Clinical and basic characteristics of Family I. **(A)** Pedigree of family I: Circles represent females, squares represent males, a slash through the symbol indicates deceased individuals, and the arrow indicates the proband. Black symbols represent patients with LS and gray symbols represent patients with tumors that have not undergone genetic testing. **(B)** Colonoscopy results of the proband: findings include colon neogenesis, surface ulceration, and easy bleeding. **(C)** The H&E staining results of the colon tissue of the proband suggest moderately differentiated adenocarcinoma. **(D–G)** Figures show strong nuclear positivity for PMS2 and MLH1, partially weak nuclear positivity for MSH6, and absence of nuclear staining for MSH2. **(H,I)** Genetic sequencing results show that the proband carries a novel *MSH2* gene mutation (NM_000251.2:c.1486delT:p.L496*), a deletion at position 1,486 in the coding region, resulting in a stop gain mutation. **(J)** The position of the p.L496* mutation in the secondary structures of the MSH2 protein. **(K)** Microsatellite instability test results. **(L,M)** Swiss-Prot predicts the protein structure of the wild-type and p.L496* MSH2 proteins. The orange region in the wild-type MSH2 protein model indicates the missing region of amino acids 496–934.

The proband from Family I was a 61-year-old man who had undergone a partial colectomy owing to a mass in the left colon, revealing a moderately differentiated adenocarcinoma. His father had died of oral cancer, his mother had died of rectal cancer at 65 years of age, his sister had been diagnosed with rectal cancer at 58 years of age, while his son was asymptomatic. Figure 1A shows the detailed pedigree of Family I, and Figure 1B shows the colonoscopy results of the proband in Family I.

**Figure 2:**
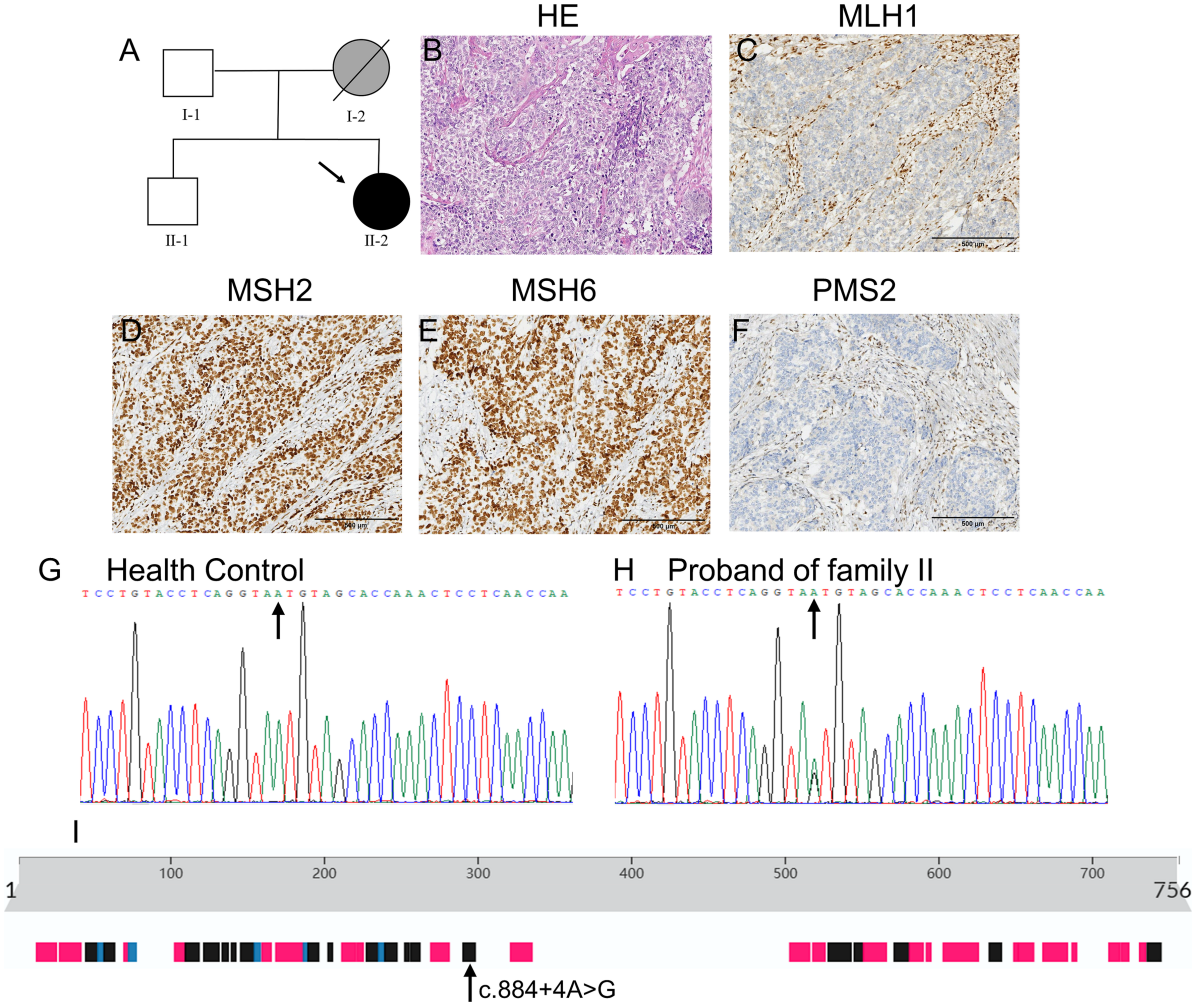
Clinical and basic characteristics of Family II. **(A)** Pedigree of family II: circles represent females, squares represent males, a slash through the symbol indicates deceased individuals, and the arrow indicates the proband. Black symbols represent patients with LS and gray symbols represent patients with tumors that have not been genetically tested. **(B)** The H&E staining results of the endometrial tissue of the proband suggest poorly differentiated endometrial adenocarcinoma. **(C–F)** Figures showed strong nuclear positivity for MSH2 and MSH6 and loss of nuclear staining for MLH1 and PMS2 in Family II. **(G,H)** Sanger sequencing results revealed that the proband in Family II carried a rare *MLH1* gene mutation (NM_001258271.1:c.884 + 4A > G). **(I)** The position of the c.884 + 4A > G mutation in the secondary structures of the MLH1 protein.

The proband of Family II was a 53-year-old woman who was diagnosed with endometrioid adenocarcinoma and underwent total hysterectomy and bilateral oophorectomy. Her mother had died of gastric cancer at the age of 68 years, and her father and brother were healthy at the time of writing. Figure 2A shows the detailed pedigree of Family II.

### Histological analysis of the tumor tissue

Paraffin-embedded tissue sections were used for histopathological analysis (hematoxylin and eosin, H&E, Figure 1C shows the H&E staining results of Family I, and Figure 2B shows the H&E staining results of Family II) and then to detect the expression of four MMR proteins: MLH1 (clone ES05), PMS2 (clone EP51), MSH2 (clone FE11), and MSH6 (clone EP49). All four primary antibodies were purchased from Jiayuan Biomedical Engineering Co., Ltd., Wuhan, China (8).

### Microsatellite instability test

Single and multiplex PCR reactions were conducted to amplify the five markers: BAT-25, MONO-27, CAT-25, BAT-26, and NR-24. The amplification reactions were carried out according to the manufacturer’s protocols.

### Whole-exome sequencing

Genomic DNA was extracted from the peripheral blood of the proband from Family I, and the tumor tissue from paraffin-embedded sections of the proband from Family II. Target regions were captured using the SureSelect Human All Exon V6 (Agilent) hybridization capture kit. High-throughput sequencing was employed using the Illumina NovaSeq (sequencing read length: 2 × 150 bp) to analyze the genomic DNA sequences (8–10). The UCSC hg19 and NCBI build 37 were used as reference genomes for WES.

### Sanger sequencing

The results of high-throughput sequencing were validated using Sanger sequencing. The polymerase chain reaction (PCR) conditions were as follows: an initial predenaturation step at 95°C for 5 min, followed by denaturation at 95°C for 30 s, annealing at 60°C for 30 s, and finally, extension at 72°C for 10 min. The PCR primers used were as follows: For Family I, the forward primer was 3′-ATAACTATTACAAGTTTTGCACA-5′ and the reverse primer was 3′-GTGACATTTAAAATAGGGCT-5′. For Family II, the forward primer was 3′-GTGACCTCACCCCTCAGGAC-5′ and the reverse primer was 3′-ACATCCTTTTGCCAGTGGTG-5′.

### Structure modeling

To construct the three-dimensional spatial structure of MSH2, SWISS-MODEL^1^ was used, and the structural differences between the wild-type and mutant MSH2 proteins were compared using the visualization software PyMOL.^2^

## Results

### Immunohistochemical analysis

Figures 1D–G show strong nuclear positivity for MLH1 (Figure 1D) and PMS2 (Figure 1G), weak nuclear positivity for MSH6 (Figure 1F), and an absence of nuclear staining for MSH2 (Figure 1E) in Family I. Figures 2C–F show strong nuclear positivity for MSH2 (Figure 2D) and MSH6 (Figure 2E) and absence of nuclear staining for MLH1 (Figure 2C) and PMS2 (Figure 2F) in Family II. The absence of MSH2, MLH1, and PMS2 expression in these patients suggests the possibility of a germline mutation in the MMR genes, indicating a high risk for LS.

### Microsatellite analysis

In Family I, as shown in Figure 1K, all markers (BAT25, MONO-27, CAT-25, BAT26, and NR-24) showed a leftward shift in the cancerous tissues compared to the normal tissues, indicating MSI in the proband’s tumor tissue.

### Genetic test results

Table 1 shows the detailed information of WES for the proband (II-2) in Family I. A novel mutation (NM_000251.2:c.1486delT:p.L496*) in the *MSH2* gene was identified (Figure 1I, the mutant position indicated by the arrow, and Figure 1H as the wild type control) in the proband (II-2). This mutation involved the deletion of thymine (T) at position 1,486 in the coding region of the *MSH2* gene, resulting in a stop gain mutation. The codon TTA encoding leucine was shifted to a stop codon TAA, leading to premature termination of the MSH2 protein translation and loss of protein integrity and function. This mutation was pathogenic for the proband and has not been previously reported.

**Table 1:** Whole-exome sequencing detail of the proband in family I and II.

Exome capture statistics	Proband I	Proband II
Total (bp)	71,945,206 (100%)	74,311,264 (100%)
Duplicate (bp)	1,069,476 (14.83%)	17,771,218 (23.91%)
Mapped (bp)	71,910,063 (99.95%)	74,271,894 (99.95%)
Properly mapped (bp)	71,333,060 (99.15%)	73,797,898 (99.31%)
PE mapped (bp)	71,882,170 (99.91%)	74,238,222 (99.90%)
SE mapped (bp)	55,785 (0.08%)	67,344 (0.09%)
Initial bases on target (bp)	60,456,963	60,456,963
Initial bases on or near target (bp)	136,297,444	136,297,444
Total effective yield (Mb)	10,741.44	11,079.83
Effective yield on target (Mb)	7,210.68	7,062.87
Fraction of effective bases on target (%)	67.1%	63.7%
Fraction of effective bases on or near target (%)	87.4%	84.9%
Average sequencing depth on target	119.27	116.82
Bases covered on target (bp)	60,324,460	60,170,790
Coverage of target region (%)	99.8%	99.5%
Fraction of target covered with at least 100× (%)	52.0%	47.0%
Fraction of target covered with at least 50× (%)	81.8%	78.5%
Fraction of target covered with at least 20× (%)	95.5%	94.6%
Fraction of target covered with at least 10× (%)	98.3%	97.8%
Fraction of target covered with at least 4× (%)	99.4%	99.1%
Gender	Male	Female

Sanger sequencing showed that the germline mutation in *MSH2* was also found in the proband’s sister (II-1), who had been diagnosed with rectal cancer at 58 years of age, but not in the proband’s son who was in good health (III-1). Given that the proband’s mother (I-2) had died of rectal cancer, we speculated that the pathogenic mutation observed in the proband was inherited from his mother.

Table 1 shows the detailed information from WES of the proband (II-2) in Family II. A heterozygous mutation (NM_001258271.1:c.884 + 4A > G) was identified in *MLH1* (Figure 2H, the mutant position indicated by the arrow, and Figure 2G as the wild type control) in the proband in Family II (II-2). This mutation causes the skipping of exon 10, producing a truncated protein, and is recognized as a pathogenic mutation (rs267607777) (11). Given that the proband’s mother (I-2) had died of gastric cancer at the age of 68 years, we speculated that this pathogenic mutation was inherited from her.

### Protein structure prediction

The three-dimensional spatial structures of the MSH2 proteins, both wild-type and the p. L496* MSH2, are shown in Figure 1L (wild-type) and Figure 1M (mutant type). The c.1486del mutation leads to the substitution of leucine at position 496 with a stop codon, resulting in the absence of amino acids 496–934 in the mutant MSH2 protein model, which leads to the loss of Domain 5 in the MSH2 protein and alterations in the sequences of Domain 3 and Domain 4 regions (12). The orange region in the wild-type MSH2 protein model is the missing region of amino acids 496–934.

## Discussion

In this study, two significant mutations were identified in two typical families with LS: one novel mutation in the *MSH2* gene (NM_000251.2:c.1486delT:p.L496*), which was not recorded in any database including GnomAD; and a previously known mutation in the *MLH1* gene (NM_001258271.1:c.884 + 4A > G), which was very rare and the mutation frequency was not recorded in any database including GnomAD. According to the American College of Medical Genetics and Genomics classification criteria, these two mutants were considered pathogenic for LS, with the *MSH2* mutant (NM_000251.2:c.1486delT:p.L496*) meeting the criteria for PVS1, PM2, PM6, and PP4, and the *MLH1* mutant (NM_001258271.1:c.884 + 4A > G) being confirmed as pathogenic.

Consistent with previous studies, immunohistochemical analysis of MMR proteins can guide the analysis of mutations in MMR genes. MLH1 combines with PMS2 to form a heterodimer, and the combined deletion of MLH1 and PMS2 is a characteristic of patients with *MLH1* mutations, while the deletion of PMS2 protein in tumor tissue indicates a germline mutation in *PMS2* (13). Similarly, loss of nuclear staining in MSH2 and MSH6 proteins indicates mutants in *MSH2* and *MSH6*, respectively (13). Our data supported these findings, with immunohistochemistry results for the proband in Family I revealing *MSH2* as the pathogenic gene and those for the proband in Family II revealing *MLH1*.

The proband in Family I showed high MSI (MSI-H). Patients with stage II colon cancer with MSI-H have a better prognosis but do not benefit from single-agent adjuvant chemotherapy with fluorouracil. Patients with colorectal cancer and MSI-H should be further tested for MMR gene mutations to confirm a diagnosis of LS. Figure 1J displays the secondary structure of the wild-type MSH2 protein, which has 934 amino acids. A variation in the *MSH2* gene (NM_000251.2:c.1486delT:p.L496*) leads to a stop gain mutation in the MSH2 protein. This mutation changes the codon TTA, which encodes leucine, to a stop codon TAA, altering the tertiary structure of the protein and eventually leading to the formation of a non-functional protein. The characteristics of the disrupted MSH2 protein may include reduced MMR activity, defective interactions with MSH6, and loss of protein expression. Given the pattern of morbidity in the family, this *MSH2* gene (NM_000251.2:c.1486delT:p.L496*) mutation was considered to be pathogenic. Therefore, the offspring of individual II-1 in Family I should undergo genetic testing. If they carry the pathogenic mutation *MSH2* (NM_000251.2:c.1486delT:p.L496*), they should undergo regular physical examinations, including gastrointestinal and gynecological endoscopy, for early diagnosis and treatment.

Furthermore, we identified a known mutation (NM_001258271.1:c.884 + 4A > G) in the *MLH1* gene that affects a donor splice site in intron 10 (Figure 2I, the mutant position in secondary structure of the wild-type MLH1 protein indicated by the arrow). Many similar *MLH1* gene variations, such as c.1668–2 A > G (14) and c.790 + 1 G > A (15), have been reported as pathogenic mutations of LS. The known mutation (NM_001258271.1:c.884 + 4A > G) in *MLH1* causes skipping of exon 10, producing a truncated protein (11). This protein is subject to nonsense-mediated decay (16), reinforcing the classification of this mutation as pathogenic. Hence, the offspring of individual II-2 in Family II should undergo genetic testing. If they carry the pathogenic *MLH1* mutation (NM_001258271.1:c.884 + 4A > G), they should undergo regular physical examinations, including gastrointestinal and gynecological endoscopy, for early diagnosis and treatment.

In 2018, Patrick summarized the strategies for surveillance, prevention, and precision medicine for managing LS-associated colorectal cancer (CRC) (17, 18). Carriers of LS should undergo colonoscopy every 1–2 years. Carriers of pathogenic *MLH1*, *MSH2*, and *MSH6* mutations can reduce their risk of developing LS-associated endometrial and ovarian cancers through preventive hysterectomy with salpingo-oophorectomy. In addition, aspirin intake reduces the risk of CRC and all LS-associated cancers. Postoperative adjuvant therapy for advanced CRC often entails the administration of 5-fluorouracil (5-FU) as a standalone treatment or in conjunction with other drugs (19). However, patients with CRC exhibiting MSI show limited response to 5-FU-based adjuvant chemotherapy. As a result, the use of 5-FU is not recommended for patients with MSI who require chemotherapy. Tumors characterized by deficient MMR pathways present ideal targets for immunotherapy, specifically, immune checkpoint inhibitors (20) targeting cytotoxic T lymphocyte antigen 4 (CTLA-4), programmed cell death 1 (PD-1), and programmed cell apoptosis ligand 1 (PD-L1), which negatively regulate T cell activation. Such patients are likely to benefit from anti-PD-1/PD-L1/CTLA-4 treatments (21–23). Ongoing trials are evaluating the effectiveness of combining chemotherapy and immunotherapy, immunotherapy and targeted therapy, and the role of immunotherapy in the adjuvant setting for patients with LS (22, 24, 25).

This study sheds light on the characteristics of LS in the Chinese population, which is of great significance for disease diagnosis, prevention, and precise treatment. The highest risk of LS-associated cancers has been linked to mutations in *MLH1* or *MSH2* (26). Previous studies have found that carriers of *MSH6* gene mutations are more likely to develop endometrial cancer, while carriers of *MSH2* gene mutations are more likely to develop extracolonic tumors or various other tumors (8). Therefore, research on the association between genotype and phenotype is crucial to provide more precise treatment strategies for patients with LS.

In summary, the study identified a novel pathogenic mutation (NM_000251.2:c.1486delT:p.L496*) in the *MSH2* gene and confirmed a known mutation in the *MLH1* gene (NM_001258271.1:c.884 + 4A > G) in two unrelated Chinese families with LS. The findings broaden our understanding of the spectrum of MMR gene mutations in China and reaffirm the importance of genetic testing for LS. Although the results of this study suggest that the novel *MSH2* mutation (NM_000251.2:c.1486delT:p.L496*) is pathogenic, further research is required to investigate its underlying pathogenic mechanisms. Genetic diagnosis, regular follow-ups, and individualized treatment should be provided to cancer-afflicted families with evidence of compromised MMR gene function.

## Data Availability

The data presented in the study are deposited in the following repository: https://www.ncbi.nlm.nih.gov/bioproject/?term=PRJNA1226963.

## Footnotes

^1^ https://swissmodel.expasy.org/

^2^ https://pymol.org/2/
